# Relationship between Rate of Hypernatremia Correction and Outcomes in Hospitalized Patients

**DOI:** 10.34067/KID.0000000785

**Published:** 2025-03-28

**Authors:** Gabriela Chacon-Palma, J. Pedro Teixeira, Igor Litvinovich, Cristian G. Bologa, Maria-Eleni Roumelioti, MingAn Yang, Mark L. Unruh

**Affiliations:** 1 Division of Nephrology, Department of Internal Medicine, University of New Mexico School of Medicine, Albuquerque, New Mexico; 2 Division of Pulmonary, Critical Care, and Sleep Medicine, University of New Mexico School of Medicine, Albuquerque, New Mexico; 3 Division of Translational Informatics, Department of Internal Medicine, University of New Mexico School of Medicine, Albuquerque, New Mexico; 4 Division of Epidemiology, Biostatistics, and Preventative Medicine, Department of Internal Medicine, University of New Mexico School of Medicine, Albuquerque, New Mexico; 5 Medicine Service, Division of Nephrology, Raymond G. Murphy VA Medical Center, Albuquerque, New Mexico

**Keywords:** clinical nephrology, electrolytes, hospitalization, hypernatremia

## Abstract

**Key Points:**

- Slow hypernatremia correction rates (≤0.50 mEq/L per hour) are associated with lower in-hospital mortality than fast rates (>0.50 mEq/L per hour).
- Fast hypernatremia correction is associated with lower odds of discharge to nursing facilities or hospice than slow correction.
- Hypernatremia severity, age, and kidney function do not modify associations between correction rates, hospital mortality, and disposition.

**Background:**

Current recommendations for limiting hypernatremia correction rates to avoid cerebral edema in adults are supported by limited evidence. We explored the associations between rate of hypernatremia correction in hospitalized adults, in-hospital mortality, and discharge disposition.

**Methods:**

Using a large, multicenter database, we analyzed 37,913 hospitalized adults with hypernatremia on admission. For the primary analysis, hypernatremia correction rates were categorized as slow (≤0.50 mEq/L per hour) or fast (>0.50 mEq/L per hour). Propensity score (PS) weighting and PS stratification were used to adjust for confounders. In secondary analyses, the results were stratified by initial sodium concentration, age, and initial eGFR. In a sensitivity analysis, correction rates were categorized as <0.40, 0.40–0.60, or >0.60 mEq/L per hour.

**Results:**

Most (89%) patients experienced slow hypernatremia correction. In PS-weighted analyses, slow correction was associated with overall lower in-hospital mortality (adjusted odds ratio [aOR], 0.63; 95% confidence interval [CI], 0.59 to 0.67) but higher odds of discharge to hospice (aOR, 1.57; 95% CI, 1.38 to 1.78) or nursing facilities (aOR, 1.60; 95% CI, 1.52 to 1.69) than fast (reference) correction rates. After stratification by initial hypernatremia severity, age, and kidney function at admission, the associations between slow versus fast correction, in-hospital mortality, and discharge disposition were largely preserved without clear signals of effect modification by subgroup. When categorizing hypernatremia correction rates into three groups, <0.40 versus >0.60 mEq/L per hour (analogous to slow versus fast, respectively) continued to be independently associated with lower in-hospital mortality but higher rates of discharge to nursing facilities or hospice.

**Conclusions:**

In this analysis, the rate of hypernatremia correction was independently associated with opposing effects on survival and a favorable discharge disposition. Our findings suggest that balancing the risks and benefits of different dysnatremia correction rates should consider not only mortality but also patient-centered outcomes such as discharge disposition.

## Visual Abstract

**Figure:**
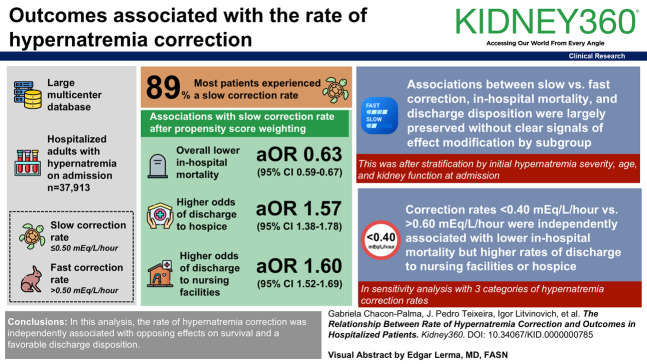

## Introduction

Hypernatremia is a common electrolyte disorder defined as a serum sodium concentration ([Na]) of >145 mEq/L.^1^ It reflects a net deficit of water as compared with total body sodium, commonly arising from losses of hypotonic fluid or free water or, less frequently, from the gain of hypertonic fluid or sodium.^2^ The prevalence of hypernatremia among all hospitalized patients ranges from 1% to 8% in most studies, with hypernatremia on admission found in a smaller subset (1%–5%).^3–6^

Hypernatremia is associated with adverse clinical outcomes,^4,7–9^ such as mortality and length of stay after adjusting for illness severity.^8,9^ Hypernatremia is also associated with discharge to a facility after hospitalization, including hospice and nursing facilities.^4^

For hypernatremia present for ≥48 hours or of unknown duration, a serum [Na] correction rate of ≤0.50 mEq/L per hour has been traditionally recommended for patients of all ages to prevent cerebral edema from overly rapid correction.^1^ This recommendation, however, is largely based on observational studies in pediatric patients.^7,10,11^ The data on treatment approaches specifically for adults are limited. Some studies have concluded that there is no increased risk from using faster correction rates.^7,11^ Other studies suggest that hospitalized adults with hypernatremia are often undertreated with inadequately slow correction and that faster correction rates are associated with improved outcomes.^10–13^

The morbidity and mortality associated with inpatient hypernatremia and the lack of consensus on the optimal rate of [Na] correction highlight the need for additional research and the establishment of evidence-based guidelines for hypernatremia management. In addition, there is a significant knowledge gap regarding the relationship between the rate of hypernatremia correction in hospitalized patients and discharge disposition, particularly when accounting for covariates that may influence this association.

To address this, we used a multicenter, national database to investigate the relationship between the rate of [Na] correction in hospitalized adults with hypernatremia on admission and clinical outcomes. These outcomes include in-hospital mortality and discharge to hospice, a nursing facility, or home. We also investigated the effect of initial hypernatremia severity, age, and kidney function on admission on these relationships.

## Methods

We conducted a retrospective observational cohort study using information from the Cerner Health Facts database on hospitalized adults with hypernatremia on admission between 2000 and 2018. The protocol (No. 19-429) and waiver for informed consent were approved by the Institutional Review Board of the University of New Mexico. The study was performed in accordance with the Declaration of Helsinki.

The index hospital admission was defined as the first inpatient encounter during the study period for patients who met the following inclusion criteria: (*1*) age 18 years or older, (*2*) first [Na] measured within 24 hours of admission >145 mEq/L, and (*3*) ≥2 [Na] measurements during the same hospitalization within 365 days from time of admission. Patients with the following characteristics were excluded from analysis: (*1*) systolic BP <90 mm Hg; (*2*) discharge disposition other than primary home, hospice, nursing facility, or in-hospital mortality; (*3*) missing eGFR values; and (*4*) failure to achieve hypernatremia correction (any reduction of [Na]) during the same hospitalization.

Correction rate was calculated as (first [Na] >145 mEq/L−first corrected [Na] ≤145 mEq/L [or last value before discharge if not fully corrected])/time in hours between the two measurements. Based on previous studies on hypernatremia correction rates and outcomes in hospitalized patients aged 18 years or older^7,13^—which have established a 0.50 mEq/L per hour rate threshold between “fast” and “slow” correction—and the aforementioned recommendations^1^ typically followed in the management of adults and children alike, we categorized correction rates as “slow” (≤0.50 mEq/L per hour) and “fast” (>0.50 mEq/L per hour) in the primary analysis. For a sensitivity analysis, we categorized correction rates as <0.40, 0.40–0.60, and >0.60 mEq/L per hour.

We discretized initial [Na] on admission into three categories: (*1*) mild hypernatremia: 146–150 mEq/L (reference), (*2*) moderate hypernatremia: 151–155 mEq/L, and (*3*) severe hypernatremia: >155 mEq/L.^13^ Kidney function was estimated using the CKD Epidemiology Collaboration equation (excluding race).^14^ We categorized initial eGFR levels into five groups: ≥90 (reference), 60–89, 30–59, 15–29, and <15 ml/min per 1.73 m^2^.

We collected patient demographics, comorbidities, leading diagnoses, laboratory values, and discharge dispositions. The International Classification of Diseases Ninth and Tenth Revisions, Clinical Modification codes, were used to identify diagnoses, and Logical Observation Identifier Names and Codes^15^ were used to identify laboratory values. We calculated the Quan-Charlson Comorbidity Index (Quan-CCI)^16^ and an adjusted version of the Sequential Organ Failure Assessment (SOFA)^17^ score at admission to adjust for comorbid conditions and illness severity, respectively. As our dataset did not provide adequately granular data to calculate all elements of the original SOFA score, we adapted the cardiovascular and respiratory subscores (Supplemental Table 1). Additionally, in concordance with the most recent consensus definition of sepsis,^18^ we assumed missing variables were normal when calculating adjusted SOFA scores.

### Statistical Analyses

Categorical variables were expressed as percentages, and continuous variables were summarized as mean±SD. Patient characteristics in the different correction rate groups were compared using Kruskal-Wallis tests for continuous variables and chi-squared tests for categorical variables.

Propensity scores (PS) were used in two ways to better adjust for potential confounders. The R Twang package was used to compute PS with age, sex, race, Quan-CCI, and adjusted SOFA score as covariates. PSs were then included in multinomial logistic regression models as weights. PS weighting (PSW) adjusts subject weights to balance covariates between treatment and control groups in observational studies, thereby mitigating bias and improving treatment effect estimates. PSW maintains the full sample size and accommodates complex models.^19^ In additional sensitivity analysis, we performed PS stratification (PSS) using the R MatchIt package.^20^ PSS is another approach for addressing differences in observed characteristics. It divides subjects into strata based on their PS, ensuring that individuals within each stratum have similar probabilities of receiving the treatment or belonging to a specific group. PSS estimates treatment effects by combining stratum-specific estimates.^21^

We evaluated interrelated associations between [Na] correction rates and discharge disposition outcomes using multinomial logistic regression models. These models accounted for competing risks of in-hospital mortality and other dispositions, with discharge to home set as the reference. For a descriptive graphical analysis, we used these models to predict probabilities of in-hospital mortality or discharge to home, hospice, or nursing facility across a continuous range of initial [Na], age, and initial eGFR. The models were adjusted for age, sex, race, Quan-CCI, and adjusted SOFA score. To assess for potential effect modification by initial [Na], age, and initial eGFR on the relationship between correction rate and discharge disposition, we added corresponding interaction terms to the models.

All statistical analyses were conducted using the R programming language (version 4.2.3).

## Results

**Figure 1:**
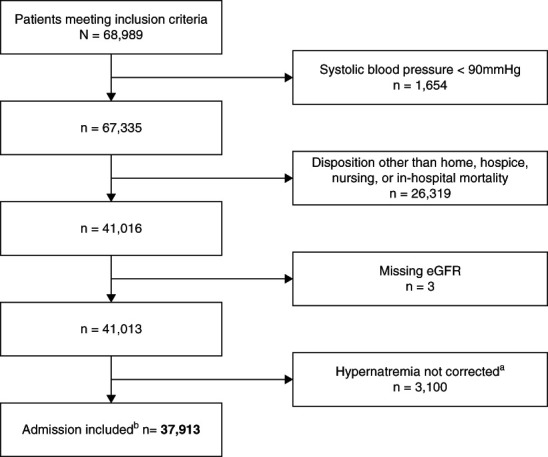
**Flow chart of the sample selection process.** ^a^ Reduction in serum [Na], of any degree, was not achieved during same hospitalization. ^b^Final cohort available for analysis. [Na], sodium concentration.

In total, 68,989 individuals met our inclusion criteria. From these, we derived a final cohort of 37,913 (Figure 1). Eighty-nine percent of patients experienced a slow [Na] correction rate, with the rest experiencing fast correction (Table 1). Notably, the in-hospital mortality among individuals with slow correction was 11% versus 18% among those with fast correction. Conversely, discharge to a nursing facility occurred in nearly double the proportion of patients (43%) who experienced slow correction compared to those with fast correction (22%). Four percent of the slow correction group was discharged to hospice as compared with 2% of the fast rate group. Of patients with fast [Na] correction, 58% were discharged home versus 44% of those with slow correction.

**Table 1:** Characteristics of hospitalized patients with hypernatremia on admission and associated sodium correction rates

Patient Characteristics	All Cohort		Fast Correction		Slow Correction	
	Counts	%	Counts	%	Counts	%
**Disposition**						
Home	17,114	45	2440	58	14,674	44
In-hospital mortality	4285	11	744	18	3541	11
Hospice	1258	3	78	2	1180	4
Nursing	15,256	40	914	22	14,342	43
eGFR						
0–14	2835	8	321	8	2514	8
15–29	5528	15	436	10	5092	15
30–59	11,125	29	901	22	10,224	30
60–89	9946	26	1050	25	8896	26
90+	8479	22	1468	35	7011	21
**Quan-CCI**						
Zero	9186	24	1207	29	7979	24
Mild	8560	23	1053	25	7507	22
Moderate	6681	18	620	15	6061	18
Severe	8232	22	624	15	7608	23
Unknown	5254	14	672	16	4582	14
**Age, yr**						
18–45	4188	11	992	24	3196	10
46–65	9180	24	1334	32	7846	23
66–75	6837	18	673	16	6164	18
76–89	13,290	35	921	22	12,369	37
90+	4418	12	256	6	4162	12
**Sex**						
Female	20,036	53	2122	51	17,914	53
**Age, yr**						
Mean/SD	70	18	61	20	71	17
**Length of stay, d**						
Mean/SD	8	10	7	11	8	10
**SOFA score^a^**						
Mean/SD	1.9	2.0	2.0	2.4	1.8	1.9

Comorbidities and Leading Diagnoses	All Cohort		Fast Correction		Slow Correction	
	Counts	%	Counts	%	Counts	%
Hypertension	12,836	34	1267	30	11,569	34
Diabetes mellitus	8648	23	854	21	7794	23
AKI	10,787	29	1025	25	9762	29
CKD 1–4	2893	8	165	4	2728	8
CKD 5	144	0.4	14	0.3	130	0.4
Ischemic heart disease	8130	21	858	21	7272	22
Heart failure	6752	18	478	12	6274	19
Peripheral vascular disease	1349	4	113	3	1236	4
Liver diseases	1843	5	289	7	1554	5
COPD	6261	17	585	14	5676	17
OB/GYN condition	107	0.3	33	0.8	74	0.2
Depression	3624	10	370	9	3254	10
Dementia	3119	8	197	5	2922	9
Head trauma	807	2	128	3	679	2
Seizure	1097	3	170	4	927	3
Cerebrovascular disease	4099	11	347	8	3752	11
Cerebral infarction	1162	3	100	2	1062	3
Cerebral hemorrhage^b^	597	2	70	2	527	2
Diabetes insipidus	207	0.6	26	0.6	181	0.5
Pyelonephritis	230	0.6	20	0.5	210	0.6
Sepsis	5737	15	526	13	5211	16
Diuretics^c^	12,039	32	1079	26	10,960	33
ICU requirement	11,545	31	1896	45	9649	29

Laboratory Values	All Cohort		Fast Correction		Slow Correction	
	Mean	SD	Mean	SD	Mean	SD
First serum [Na], mEq/L	150	6	151	6	150	6
Serum albumin, g/dl	3.4	0.8	3.5	0.9	3.4	0.8
Anion gap, mmol/L	13	6	16	8	13	5
BUN, mmol/L	40	33	32	32	41	33
Phosphorus, mg/dl	3.8	1.8	4.1	2.4	3.7	1.6
Magnesium, mmol/L	2.2	0.6	2.1	0.7	2.2	0.5
Chloride, mmol/L	111	9	111	10	111	9
Serum potassium, mEq/L	4.1	0.8	4.1	1.0	4	0.8
CO_2_, mEq/L	25	5.9	23	6.6	25	5.7
Serum creatinine, mg/dl	1.70	1.76	1.67	2.06	1.71	1.72
Glucose, mg/dl	157	125	162	134	156	124
Hemoglobin, g/dl	12.4	2.6	12.7	2.8	12.3	2.6
Serum total protein, mg/dl	6.8	1.1	6.6	1.4	6.8	1.1
ALT, IU/L	47	100	66	149	45	93
AST, IU/L	58	122	87	185	54	111
eGFR, ml/min per 1.73 m^2^	61	33	71	36	59	32

Fast correction: >0.50 mEq/L per hour; slow correction: ≤0.50 mEq/L per hour. ALT, alanine aminotransferase; AST, aspartate aminotransferase; CO_2_, carbon dioxide; COPD, chronic obstructive pulmonary disease; ICU, intensive care unit; IU, international units; [Na], sodium concentration; OB/GYN, obstetrics/gynecology; Quan-CCI, Quan-Charlson comorbidity index; SOFA, Sequential Organ Failure Assessment.

a Adjusted SOFA score outlined in Supplemental Table 1.

b Includes subarachnoid, subdural, and nontraumatic intracerebral hemorrhage.

c Diuretics: chlorthalidone, chlorothiazide, hydrochlorothiazide, indapamide, metolazone, bumetanide, ethacrynic acid, furosemide, torsemide, amiloride, eplerenone, spironolactone, and triamterene.

Patient and clinical characteristics, including demographics, leading diagnoses, comorbidities, and laboratory values, are presented in Table 1. Patients receiving slow correction were on average 10 years older and had a mean eGFR 12 ml/min per 1.73 m^2^ lower than individuals receiving fast correction. Both groups had similar mean adjusted SOFA scores and first [Na] levels.

**Figure 2:**
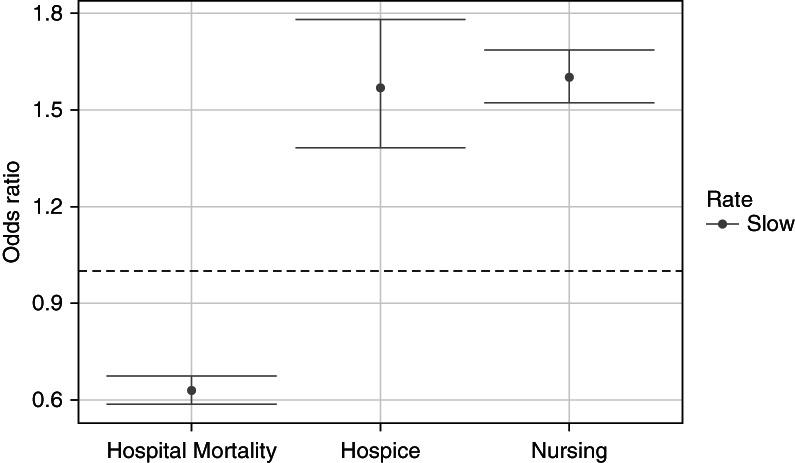
**Plots of aORs (95% CI) for in-hospital mortality and discharge to hospice or a nursing facility associated with a slow sodium correction rate relative to fast correction.** Slow: ≤0.50 mEq/L per hour; fast: >0.50 mEq/L per hour (reference). aORs derived from a multinomial logistic regression model adjusted for age, sex, race, Quan-CCI, and adjusted SOFA score. Model includes PSs as weights. aOR, adjusted odds ratio; CI, confidence interval; PS, propensity score; Quan-CCI, Quan-Charlson Comorbidity Index; SOFA, Sequential Organ Failure Assessment.

One of the most significant findings of this study was that after PSW, the overall adjusted odds ratio (aOR) of in-hospital mortality was lower with slow [Na] correction (aOR, 0.63; 95% confidence interval [CI], 0.59 to 0.67) than with fast (reference) correction (Figure 2 and Table 2). By contrast, the aORs of discharge to hospice (1.57; 95% CI, 1.38 to 1.78) or a nursing facility (1.60; 95% CI, 1.52 to 1.69) were higher with slow correction. When examining these associations using PSS, the results were largely similar. In the sensitivity analysis with three correction rate categories, the associations between rates <0.40 mEq/L per hour versus >0.60 mEq/L per hour (analogous to slow versus fast, respectively), in-hospital morality, and discharge to a facility were preserved (Table 3).

**Table 2:** Relationship between sodium correction rate (slow versus fast) and disposition outcomes

Correction Rate	In-Hospital Mortality (*n*=4285)	Discharge to Hospice (*n*=1258)	Discharge to Nursing Facility (*n*=15,256)
	PS Weighted	PS Weighted	PS Weighted
	aOR (95% CI)	aOR (95% CI)	aOR (95% CI)
Slow	0.63 (0.59 to 0.67)	1.57 (1.38 to 1.78)	1.60 (1.52 to 1.69)
Fast	Reference	Reference	Reference

Slow: ≤0.50 mEq/L per hour; fast: >0.50 mEq/L per hour. aORs derived from a multinomial logistic regression model adjusted for age, sex, race, Quan-Charlson Comorbidity Index, and adjusted Sequential Organ Failure Assessment score. PSs included in model as weights. aOR, adjusted odds ratio; CI, confidence interval; PS, propensity score.

**Table 3:** Relationship between sodium correction rate (<0.40 versus 0.40–0.60 versus >0.60) and disposition outcomes

Correction Rate	In-Hospital Mortality (*n*=4285)	Discharge to Hospice (*n*=1258)	Discharge to Nursing Facility (*n*=15,256)
	PS Weighted	PS Weighted	PS Weighted
	aOR (95% CI)	aOR (95% CI)	aOR (95% CI)
<0.40	0.56 (0.51 to 0.61)	1.35 (1.16 to 1.56)	1.80 (1.69 to 1.92)
0.40–0.60	0.65 (0.60 to 0.70)	1.05 (0.90 to 1.22)	1.54 (1.44 to 1.64)
>0.60	Reference	Reference	Reference

Sodium correction rates in mEq/L per hour. aORs derived from a multinomial logistic regression model adjusted for age, sex, race, Quan-Charlson Comorbidity Index, and adjusted Sequential Organ Failure Assessment score. PSs included in model as weights. aOR, adjusted odds ratio; CI, confidence interval; PS, propensity score.

### Initial [Na] and Outcomes

**Figure 3:**
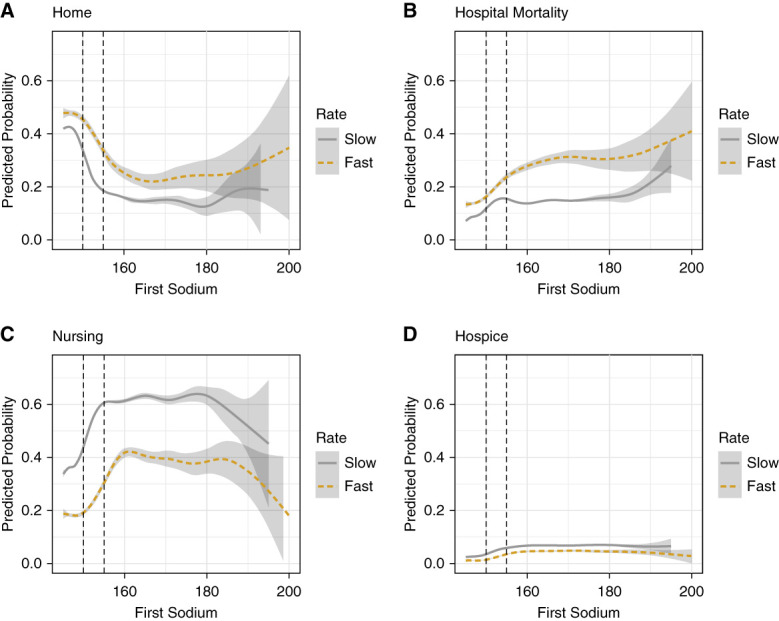
**Crude probability of different discharge dispositions as a function of first serum [Na] on admission stratified by sodium correction rate.** Discharge to (A) home, (B) in-hospital mortality, (C) nursing facility, and (D) hospice. Predicted probabilities derived from a multinomial logistic regression model. Slow: ≤0.50 mEq/L per hour; fast: >0.50 mEq/L per hour. First [Na] in mEq/L.

When stratifying by first [Na], in general, as [Na] increased, the crude probability of being discharged home decreased and that of in-hospital mortality and of discharge to a nursing facility or hospice increased until reaching a plateau at approximately 160 mEq/L (Figure 3). When compared with patients who experienced fast [Na] correction rates, patients with slow rates had a lower probability of in-hospital mortality (Figure 3B) but also a lower likelihood of being discharged home (Figure 3A) across all first [Na]. Irrespective of hypernatremia severity, the fast correction group had a significantly lower probability of being discharged to a nursing facility (Figure 3C) and a marginally smaller likelihood of discharge to hospice (Figure 3D). Supplemental Figure 1 depicts the distribution of initial serum [Na] in the final cohort.

**Figure 4:**
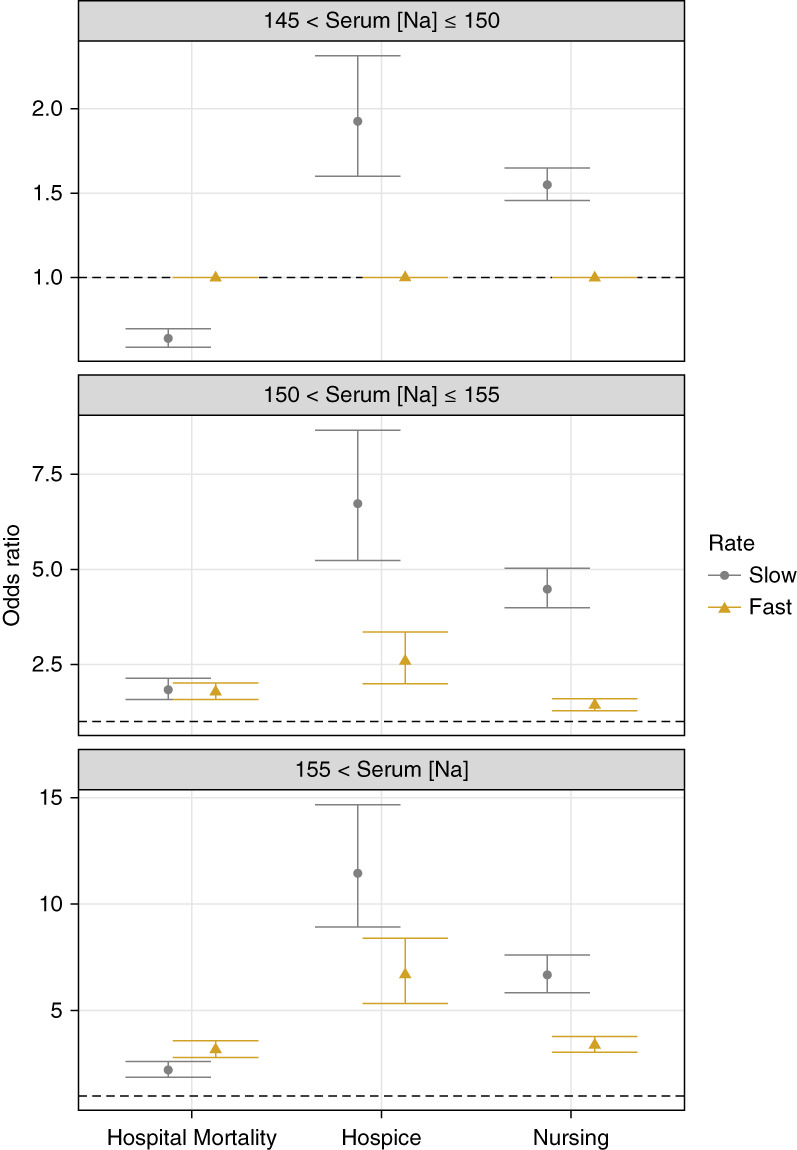
**Plots of aORs (95% CI) for in-hospital mortality and discharge to hospice or a nursing facility associated with different sodium correction rates stratified by first [Na] level on admission.** Slow: ≤0.50 mEq/L per hour; fast: >0.50 mEq/L per hour. aORs derived from a multinomial logistic regression model adjusted for age, sex, race, Quan-CCI, and adjusted SOFA score. Model includes PSs as weights.

The most significant findings of this secondary analysis are presented on Figure 4 and Table 4. After PSW, the slow correction group had significantly lower aORs of in-hospital mortality when hypernatremia was mild (slow: aOR 0.64; 95% CI, 0.59 to 0.70; fast: reference) or severe (slow: aOR, 2.22; 95% CI, 1.87 to 2.62; fast: aOR, 3.18; 95% CI, 2.81 to 3.60). Hospital mortality aORs were not significantly different with moderate hypernatremia. When compared with slow correction, aORs of discharge to hospice or a nursing facility were significantly lower for subjects with fast correction in all hypernatremia severity subgroups. Similar aORs were obtained after PSS. In the three-category sensitivity analysis, except for the nonsignificant differences between aORs for discharge to hospice observed in the severe hypernatremia subgroup, the associations between correction rates <0.40 versus >0.60 mEq/L per hour (analogous to slow versus fast, respectively), in-hospital mortality, and discharge to a facility remained unchanged (Table 5).

**Table 4:** Relationship between sodium correction rate (slow versus fast) and disposition outcomes stratified by hypernatremia severity on admission

Hypernatremia Severity	Correction Rate	In-Hospital Mortality (*n*=4285)	Discharge to Hospice (*n*=1258)	Discharge to Nursing Facility (*n*=15,256)
		PS Weighted	PS Weighted	PS Weighted
		aOR (95% CI)	aOR (95% CI)	aOR (95% CI)
Mild	Slow	0.64 (0.59 to 0.70)	1.93 (1.60 to 2.31)	1.55 (1.46 to 1.65)
	Fast	Reference	Reference	Reference
Moderate	Slow	1.84 (1.58 to 2.14)	6.73 (5.23 to 8.66)	4.48 (3.99 to 5.03)
	Fast	1.78 (1.58 to 2.01)	2.59 (2.00 to 3.35)	1.43 (1.29 to 1.59)
Severe	Slow	2.22 (1.87 to 2.62)	11.44 (8.93 to 14.67)	6.67 (5.85 to 7.62)
	Fast	3.18 (2.81 to 3.60)	6.69 (5.33 to 8.39)	3.40 (3.05 to 3.79)

Mild hypernatremia: sodium concentration=146–150 mEq/L; moderate hypernatremia: sodium concentration=151–155 mEq/L; severe hypernatremia: sodium concentration=155+ mEq/L. Slow: ≤0.50 mEq/L per hour; fast: >0.50 mEq/L per hour. aORs derived from a multinomial logistic regression model adjusted for age, sex, race, Quan-Charlson Comorbidity Index, and adjusted Sequential Organ Failure Assessment score. PSs included in model as weights. aOR, adjusted odds ratio; CI, confidence interval; PS, propensity score.

**Table 5:** Relationship between sodium correction rate (<0.40 versus 0.40–0.60 versus >0.60) and disposition outcomes stratified by hypernatremia severity on admission

Hypernatremia Severity	Correction Rate	In-Hospital Mortality (*n*=4285)	Discharge to Hospice (*n*=1258)	Discharge to Nursing Facility (*n*=15,256)
		PS Weighted	PS Weighted	PS Weighted
		aOR (95% CI)	aOR (95% CI)	aOR (95% CI)
Mild	<0.40	0.55 (0.49 to 0.61)	1.73 (1.39 to 2.14)	1.61 (1.49 to 1.74)
	0.40–0.60	0.57 (0.51 to 0.63)	0.89 (0.68 to 1.14)	1.14 (1.05 to 1.23)
	>0.60	Reference	Reference	Reference
Moderate	<0.40	1.60 (1.33 to 1.92)	6.16 (4.54 to 8.35)	4.83 (4.17 to 5.58)
	0.40–0.60	1.43 (1.23 to 1.67)	2.50 (1.83 to 3.41)	2.50 (2.20 to 2.83)
	>0.60	1.48 (1.28 to 1.70)	2.86 (2.16 to 3.79)	1.21 (1.06 to 1.38)
Severe	<0.40	1.89 (1.53 to 2.33)	9.95 (7.35 to 13.47)	6.98 (5.90 to 8.25)
	0.40–0.60	1.98 (1.69 to 2.33)	9.17 (7.14 to 11.78)	6.30 (5.54 to 7.16)
	>0.60	2.96 (2.55 to 3.43)	6.59 (5.08 to 8.54)	2.72 (2.37 to 3.13)

Mild hypernatremia: sodium concentration=146–150 mEq/L; moderate hypernatremia: sodium concentration=151–155 mEq/L; severe hypernatremia: sodium concentration=155+ mEq/L. Sodium correction rates in mEq/L per hour. aORs derived from a multinomial logistic regression model adjusted for age, sex, race, Quan-Charlson Comorbidity Index, and adjusted Sequential Organ Failure Assessment score. PSs included in model as weights. aOR, adjusted odds ratio; CI, confidence interval; PS, propensity score.

### Age and Outcomes

In general, for ages 40 or older, the probability of discharge to home (Supplemental Figure 2A) decreased and that of in-hospital mortality (Supplemental Figure 2B) and of discharge to a nursing facility (Supplemental Figure 2C) increased with increasing age. The probabilities of discharge to hospice (Supplemental Figure 2D) were similar for fast and slow correction irrespective of age, with small increments after age 60. Patients of all ages with slow correction had a lower probability of in-hospital mortality than those with fast correction. This difference in probability of in-hospital mortality between the two groups increased after the age of approximately 60. Of the patients who survived to discharge, those 45 years or older with fast correction had a marginally greater probability of being discharged home. Subjects of all ages who experienced a fast correction rate had a lower likelihood of discharge to a nursing facility.

Importantly, we observed that patients of all ages with slow correction (versus fast) had significantly lower aORs of in-hospital mortality in the PS-weighted analysis (Supplemental Figure 3 and Supplemental Table 2). Adjusted odds for discharge to hospice or a nursing facility were higher with slow correction in every age subgroup. However, differences in aORs between fast and slow correction were statistically nonsignificant for discharge to hospice in all age subgroups and for discharge to a nursing facility in those aged 76–89 years. Similar aORs were computed after PSS.

### eGFR and Outcomes

After stratification by the initial kidney function level, the crude probability of being discharged home (Supplemental Figure 4A) increased with increasing eGFR values ≥20 ml/min per 1.73 m^2^ for both correction rate groups. The probability of in-hospital mortality (Supplemental Figure 4B) declined with increasing eGFR until reaching a plateau at approximately 100 ml/min per 1.73 m^2^. With decreasing eGFR, the likelihood of discharge to a nursing facility (Supplemental Figure 4C) increased until reaching approximately 20 ml/min per 1.73 m^2^ and then steadily declined. The probabilities of being discharged to hospice (Supplemental Figure 4D) were nearly identical for fast and slow correction across eGFR levels. When comparing the two correction rates, the likelihood of in-hospital mortality was lower with slow correction up to eGFR 100 ml/min per 1.73 m^2^; thereafter, the curves followed similar trajectories. Of the patients surviving to discharge, irrespective of eGFR, those with fast correction had a higher probability of being discharged home and a lower probability of discharge to a nursing facility.

Most importantly for this secondary analysis, we found that after PSW, slow correction (versus fast) was associated with significantly lower adjusted odds of in-hospital mortality in all but one (60–89 ml/min per 1.73 m^2^ subgroup) of the eGFR categories (Supplemental Figure 5 and Supplemental Table 3). The aOR differences for discharge to hospice were all nonsignificant except with eGFR ≥90 ml/min per 1.73 m^2^; these patients had lower odds of discharge to hospice with fast correction. Subjects with fast correction also had lower aORs of discharge to a nursing facility across all kidney function levels. The aORs derived from PS-stratified analysis were similar in value.

## Discussion

In this large, multicenter database study, we investigated the relationship between the rate of [Na] correction, in-hospital mortality, and discharge disposition among nearly 38,000 hospitalized adults with hypernatremia on admission. Consistent with previous studies, most of our cohort (89%) experienced slow [Na] correction; however, unlike other investigations, we observed that slow correction was beneficial for mortality.^7,13^

Somewhat unexpectedly, the rate of hypernatremia correction had opposite associations with survival and with rates of a favorable discharge disposition. Specifically, when compared with patients with fast correction rates, a lower percentage of subjects with slow correction suffered in-hospital mortality or were discharged home. However, a higher percentage of patients with slow correction were discharged to nursing facilities or hospice. Similarly, in logistic regression models with PSW, slow correction was independently associated with overall lower adjusted odds of in-hospital mortality and with increased adjusted odds of discharge to hospice or a nursing facility than fast correction. The sensitivity analysis incorporating three correction rate categories reproduced these results. The associations between rates <0.40 versus >0.60 mEq/L per hour (analogous to slow versus fast, respectively), in-hospital mortality, and discharge to a facility were preserved.

We compared our findings to those of two retrospective cohort studies investigating the association between [Na] correction rate and 30-day mortality in hospitalized adults with severe hypernatremia ([Na] >155 mEq/L). Both defined fast correction as >0.50 mEq/L per hour and slow as ≤0.50 mEq/L per hour. Chauhan *et al.*^7^ found no significant difference in 30-day mortality between the two correction rates, whereas Feigin *et al.*^13^ observed that slow correction was associated with higher 30-day mortality than fast correction. Another study of hospitalized patients with severe hypernatremia found that correction rates ≥0.25 mEq/L per hour (fast) in the first 24 hours after admission were associated with lower 30-day mortality than rates <0.25 mEq/L per hour (slow).^12^ Darmon *et al.*^22^ observed a median [Na] correction rate of 2.44 mmol/L per day, equivalent to 0.10 mEq/L per hour, in intensive care unit patients with hypernatremia on day 1. They observed that faster correction during the first 3 days was associated with lower 28-day mortality. Although these studies found that faster correction was either more favorable or no different than slower correction for reducing mortality, there are important differences between these investigations and our analysis. Correction speeds of 0.25 and 0.10 mEq/L per hour are markedly lower than our threshold separating fast and slow correction. Although our definitions of fast and slow correction in the primary analysis match those selected by Chauhan *et al.*^7^ and Feigin *et al.*,^13^ our studies differ in illness severity of the cohort and the period observed for mortality. Unlike the present investigation, their analyses were limited to critically ill patients and/or those with severe hypernatremia. In addition, we examined in-hospital mortality rather than 4-week mortality, which may account for discrepancies.

One mechanism that may explain the association between fast hypernatremia correction and higher in-hospital mortality is the risk of neurologic sequelae, particularly cerebral edema and seizures, arising from rapid lowering of serum [Na]. Notwithstanding, we acknowledge that this phenomenon has thus far only been documented in the pediatric population. Another possible mechanism is the aggressive administration of dilute fluids to lower serum [Na]. Although fluid without sodium is traditionally felt to contribute less to fluid overload, fast correction rates can lead to the administration of large volumes of fluid and may result in fluid overload, which has been strongly associated with in-hospital mortality.^23^

On stratification, in general, the crude probability of in-hospital mortality and discharge to hospice or a nursing facility increased and the incidence of home discharges decreased with increasing initial [Na], worsening initial kidney function, and older age. These findings were mostly consistent with a previous study^4^; however, the increased likelihood of being discharged home when eGFR fell below approximately 20 ml/min per 1.73 m^2^ was unexpected. One possible explanation is that the initiation of maintenance dialysis in patients with ESKD may facilitate home discharges. Interestingly, we noted a plateau for initial serum [Na] values ≥160 mEq/L in Figure 3, although the relatively small number of patients in that range produced wide CIs. Nonetheless, a possible explanation for this plateau is that this degree of hypernatremia may be associated with such high severity of illness that rate of [Na] correction no longer significantly affects outcomes.

We largely failed to detect any significant effect modification by initial hypernatremia severity, age, or initial kidney function on the associations between rates of hypernatremia correction and outcomes. Consistent with our primary analysis, patients with slow (versus fast) correction rates had a lower probability of in-hospital mortality but also a lower likelihood of being discharged home across levels of first [Na], age, and initial eGFR. These findings were largely preserved in the logistic regression analysis. Slow (versus fast) and <0.40 mEq/L per hour (versus >0.60 mEq/L per hour) correction rates remained independently associated with significantly lower in-hospital mortality but also with increased adjusted odds of discharge to a nursing facility across most subgroups. However, the relationships between [Na] correction rate, in-hospital mortality, and discharge disposition were attenuated in some subgroups, particularly for hospice discharge.

Our findings suggest that the relationships between rate of hypernatremia correction, survival, and a favorable disposition are complex and are likely affected by competing risks of in-hospital mortality and discharge to a facility. A similar phenomenon has been illustrated in randomized controlled trials conducted in the setting of cardiac arrest, which have demonstrated that interventions can result in improved survival at the expense of worsening functional status or neurologic outcomes.^24,25^

This report has several strengths. A notable feature is the use of a large, diverse database comprised of information from hundreds of centers across the United States and encompassing varying levels of acuity and degrees of hypernatremia. Second, this study shows the relationship between rate of hypernatremia correction and discharge disposition outcomes other than mortality. We also addressed a knowledge gap by stratifying the relationship between [Na] correction rate and outcomes by hypernatremia severity, age, and eGFR level. In addition, our analysis incorporated PS to reduce confounding and enhance the validity of our results. Finally, we performed a sensitivity analysis with three categories of correction rates and observed that the associations between rates >0.60 versus <0.40 mEq/L per hour (analogous to fast versus slow, respectively) and outcomes were consistent, strengthening the robustness of our results.

There are limitations to this study. Despite controlling for a variety of important confounders in multiple ways, the retrospective nature of the analysis precludes making conclusions about causation. Second, we were unable to analyze postdischarge outcomes such as 30-mortality or rehospitalization due to the limited longitudinal information available in the database. Third, we did not adjust serum [Na] for blood glucose levels >100 mg/dl due to the large number of patients who did not have both levels measured simultaneously. Including only patients with both levels measured simultaneously would have significantly diminished our cohort size. Although lack of [Na] adjustment for elevated glucose may have affected categorization into hypernatremia severity subgroups or calculated correction rates, this effect was likely modest. Based on the cohort's mean glucose concentration of 157 mg/dl, a [Na] correction factor of 1.6–2.4 mEq/L^26,27^ would lead to a 1 mEq/L higher mean initial [Na] (mean first [Na]=150 mEq/L). Furthermore, only very high or drastically changed glucose values would affect the numerator of the formula used to compute correction rates.

Our study addresses knowledge gaps regarding the significance of hypernatremia correction rates among hospitalized adults by including patient outcomes previously unexplored in this context and through the incorporation of key covariates into the analysis. This large cohort study suggests that although slow correction rates may be associated with lower in-hospital mortality, the potential for competing risks of survival and a favorable disposition may translate into higher rates of discharge to facilities with slow correction. This underscores the importance of considering functional status, in addition to survival, when balancing the risks and benefits of employing different [Na] correction rates. Further studies in adults are needed to more clearly delineate the multi-faceted relationship between rate of hypernatremia correction and clinical outcomes. Future investigations, ideally in the form of randomized controlled trials, will help reconcile discrepant conclusions in the literature to better inform treatment guidelines.

## Supplementary Material

## Data Sharing Statement

Data cannot be shared. Although data from Cerner Health Facts is de-identified, it is still considered confidential patient data. The Health Facts user agreement indicates that raw data from Health Facts may not be shared with others and may only be used by authorized individuals for job-related tasks.

## Supplemental Material

Supplemental Table 1. Adjusted SOFA score. Patients with noninvasive mechanical ventilation include only those with acute (±chronic) respiratory failure or distress. MAP, mean arterial pressure.

Supplemental Table 2. Relationship between sodium correction rate and disposition outcomes stratified by age. Slow: ≤0.50 mEq/L per hour; fast: >0.50 mEq/L per hour. aORs derived from a multinomial logistic regression model adjusted for age, sex, race, Quan-CCI, and adjusted SOFA score. PS included in model as weights.

Supplemental Table 3. Relationship between sodium correction rate and disposition outcomes stratified by eGFR. Slow: ≤0.50 mEq/L per hour; fast: >0.50 mEq/L per hour. aORs derived from a multinomial logistic regression model adjusted for age, sex, race, Quan-CCI, and adjusted SOFA score. PS included in model as weights.

Supplemental Figure 1. Distribution of initial serum [Na] in the final cohort. [Na] in mEq/L.

Supplemental Figure 2. Crude probability of discharge to home (A), in-hospital mortality (B), nursing facility (C), and hospice (D) as a function of age stratified by sodium correction rate. Predicted probabilities derived from a multinomial logistic regression model. Slow: ≤0.50 mEq/L per hour; fast: >0.50 mEq/L per hour.

Supplemental Figure 3. Plots of aORs (95% CI) for in-hospital mortality and discharge to hospice or a nursing facility associated with different sodium correction rates stratified by age. Slow: ≤0.50 mEq/L per hour; fast: >0.50 mEq/L per hour. aORs derived from a multinomial logistic regression model adjusted for age, sex, race, Quan-CCI, and adjusted SOFA score. Model includes PSs as weights. ≤ denotes ≤.

Supplemental Figure 4. Crude probability of discharge to home (A), in-hospital mortality (B), nursing facility (C), and hospice (D) as a function of eGFR stratified by sodium correction rate. Predicted probabilities derived from a multinomial logistic regression model. Slow: ≤0.50 mEq/L per hour; fast: >0.50 mEq/L per hour.

Supplemental Figure 5. Plots of aORs (95% CI) for in-hospital mortality and discharge to hospice or a nursing facility associated with different sodium correction rates stratified by eGFR level. Slow: ≤0.50 mEq/L per hour; fast: >0.50 mEq/L per hour aORs derived from a multinomial logistic regression model adjusted for age, sex, race, Quan-CCI, and adjusted SOFA score. Model includes PSs as weights. ≤ denotes ≤.
