# Supplementary material for: Relationship between Rate of Hypernatremia Correction and Outcomes in Hospitalized Patients
Source: Kidney360. 2025 Mar 28;6(8):1305–16. doi: 10.34067/KID.0000000785 (PMC12407122; doi:10.34067/KID.0000000785)
Supplement: Supplementary file 1 [file kidney360-6-1305-s001.pdf]

ASN American Society of Nephrology | JASN Journal of the American Society of Nephrology | KIDNEY360 Accessing Our World From Every Angle | CJASN Clinical Journal of the American Society of Nephrology

ASN Journal Disclosure Form

As per ASN journal policy, I have disclosed any financial relationships or commitments I have held in the past 36 months as included below. I have listed my Current Employer below to indicate there is a relationship requiring disclosure. If no relationship exists, my Current Employer is not listed.

C. Bologa reports the following:
Ownership Interest: Alakai Health; Research Funding: Givaudan Flavors; and Patents or Royalties: Linaeus Therapeutics.

I understand that the information above will be published within the journal article, if accepted, and that failure to comply and/or to accurately and completely report the potential financial conflicts of interest could lead to the following: 1) Prior to publication, article rejection, or 2) Post-publication, sanctions ranging from, but not limited to, issuing a correction, reporting the inaccurate information to the authors' institution, banning authors from submitting work to ASN journals for varying lengths of time, and/or retraction of the published work.

Name: Cristian George Bologa
Manuscript ID: K360-2024-001141R1
Manuscript Title: The Relationship between Rate of Hypernatremia Correction and Outcomes in Hospitalized Patients
Date of Completion: March 14, 2025
Disclosure Updated Date: March 14, 2025

ASN American Society of Nephrology JASN Journal of the American Society of Nephrology KIDNEY360 Accessing Our World From Every Angle CJASN Clinical Journal of the American Society of Nephrology

ASN Journal Disclosure Form

As per ASN journal policy, I have disclosed any financial relationships or commitments I have held in the past 36 months as included below. I have listed my Current Employer below to indicate there is a relationship requiring disclosure. If no relationship exists, my Current Employer is not listed.

G. Chacon-Palma reports the following:
Employer: University of New Mexico School of Medicine

I understand that the information above will be published within the journal article, if accepted, and that failure to comply and/or to accurately and completely report the potential financial conflicts of interest could lead to the following: 1) Prior to publication, article rejection, or 2) Post-publication, sanctions ranging from, but not limited to, issuing a correction, reporting the inaccurate information to the authors' institution, banning authors from submitting work to ASN journals for varying lengths of time, and/or retraction of the published work.

Name: Gabriela Chacon-Palma
Manuscript ID: K360-2024-001141R1
Manuscript Title: The Relationship between Rate of Hypernatremia Correction and Outcomes in Hospitalized Patients
Date of Completion: March 14, 2025
Disclosure Updated Date: March 14, 2025

ASN Journal Disclosure Form

As per ASN journal policy, I have disclosed any financial relationships or commitments I have held in the past 36 months as included below. I have listed my Current Employer below to indicate there is a relationship requiring disclosure. If no relationship exists, my Current Employer is not listed.

I. Litvinovich reports the following:
Employer: The University of New Mexico

I understand that the information above will be published within the journal article, if accepted, and that failure to comply and/or to accurately and completely report the potential financial conflicts of interest could lead to the following: 1) Prior to publication, article rejection, or 2) Post-publication, sanctions ranging from, but not limited to, issuing a correction, reporting the inaccurate information to the authors' institution, banning authors from submitting work to ASN journals for varying lengths of time, and/or retraction of the published work.

Name: Igor Litvinovich
Manuscript ID: K360-2024-001141R1
Manuscript Title: The Relationship between Rate of Hypernatremia Correction and Outcomes in Hospitalized Patients
Date of Completion: March 13, 2025
Disclosure Updated Date: March 13, 2025

ASN American Society of Nephrology
JASN Journal of the American Society of Nephrology
KIDNEY360 Accessing Our World From Every Angle
CJASN Clinical Journal of the American Society of Nephrology

ASN Journal Disclosure Form

As per ASN journal policy, I have disclosed any financial relationships or commitments I have held in the past 36 months as included below. I have listed my Current Employer below to indicate there is a relationship requiring disclosure. If no relationship exists, my Current Employer is not listed.

M. Roumelioti reports the following:
Employer: University of New Mexico; Consultancy: My spouse: Quanta; Advisory or Leadership Role: Chair of the Medical Board ESRD Network 13; and Other Interests or Relationships: Participating in DCI quality meetings and receiving financial support.

I understand that the information above will be published within the journal article, if accepted, and that failure to comply and/or to accurately and completely report the potential financial conflicts of interest could lead to the following: 1) Prior to publication, article rejection, or 2) Post-publication, sanctions ranging from, but not limited to, issuing a correction, reporting the inaccurate information to the authors' institution, banning authors from submitting work to ASN journals for varying lengths of time, and/or retraction of the published work.

Name: Maria-Eleni Roumelioti
Manuscript ID: K360-2024-001141R1
Manuscript Title: The Relationship between Rate of Hypernatremia Correction and Outcomes in Hospitalized Patients
Date of Completion: March 11, 2025
Disclosure Updated Date: March 11, 2025

ASN American Society of Nephrology | JASN Journal of the American Society of Nephrology | KIDNEY360 Accessing Our World From Every Angle | CJASN Clinical Journal of the American Society of Nephrology

ASN Journal Disclosure Form

As per ASN journal policy, I have disclosed any financial relationships or commitments I have held in the past 36 months as included below. I have listed my Current Employer below to indicate there is a relationship requiring disclosure. If no relationship exists, my Current Employer is not listed.

J. Teixeira reports the following:
Consultancy: Outset Medical; Ownership Interest: Eli Lilly and Company, Novo Nordisk A/S, Pfizer Inc.; Research Funding: Sentien Biotechnologies Inc.; Rediscovery Life Sciences LLC; Gilead; La Jolla Pharmaceutical Company/Innoviva; Astute Medical/bioMérieux; Honoraria: Outset Medical; and Speakers Bureau: Outset Medical.

I understand that the information above will be published within the journal article, if accepted, and that failure to comply and/or to accurately and completely report the potential financial conflicts of interest could lead to the following: 1) Prior to publication, article rejection, or 2) Post-publication, sanctions ranging from, but not limited to, issuing a correction, reporting the inaccurate information to the authors' institution, banning authors from submitting work to ASN journals for varying lengths of time, and/or retraction of the published work.

Name: J. Pedro Teixeira
Manuscript ID: K360-2024-001141R1
Manuscript Title: The Relationship between Rate of Hypernatremia Correction and Outcomes in Hospitalized Patients
Date of Completion: March 12, 2025
Disclosure Updated Date: March 12, 2025

ASN
American Society of Nephrology
JASN
Journal of the American Society of Nephrology
KIDNEY360
Accessing Our World From Every Angle
CJASN
Clinical Journal of the American Society of Nephrology

ASN Journal Disclosure Form

As per ASN journal policy, I have disclosed any financial relationships or commitments I have held in the past 36 months as included below. I have listed my Current Employer below to indicate there is a relationship requiring disclosure. If no relationship exists, my Current Employer is not listed.

M. Unruh reports the following:
Employer: University of New Mexico; New Mexico Veterans Hospital; and Research Funding: Dialysis Clinic Inc.

I understand that the information above will be published within the journal article, if accepted, and that failure to comply and/or to accurately and completely report the potential financial conflicts of interest could lead to the following: 1) Prior to publication, article rejection, or 2) Post-publication, sanctions ranging from, but not limited to, issuing a correction, reporting the inaccurate information to the authors' institution, banning authors from submitting work to ASN journals for varying lengths of time, and/or retraction of the published work.

Name: Mark L. Unruh
Manuscript ID: K360-2024-001141R1
Manuscript Title: The Relationship between Rate of Hypernatremia Correction and Outcomes in Hospitalized Patients
Date of Completion: March 15, 2025
Disclosure Updated Date: September 7, 2024

ASN American Society of Nephrology | JASN Journal of the American Society of Nephrology | KIDNEY360 Accessing Our World From Every Angle | CJASN Clinical Journal of the American Society of Nephrology

ASN Journal Disclosure Form

As per ASN journal policy, I have disclosed any financial relationships or commitments I have held in the past 36 months as included below. I have listed my Current Employer below to indicate there is a relationship requiring disclosure. If no relationship exists, my Current Employer is not listed.

M. Yang reports the following:
Employer: UNM

I understand that the information above will be published within the journal article, if accepted, and that failure to comply and/or to accurately and completely report the potential financial conflicts of interest could lead to the following: 1) Prior to publication, article rejection, or 2) Post-publication, sanctions ranging from, but not limited to, issuing a correction, reporting the inaccurate information to the authors' institution, banning authors from submitting work to ASN journals for varying lengths of time, and/or retraction of the published work.

Name: Mingan Yang
Manuscript ID: K360-2024-001141R1
Manuscript Title: The Relationship between Rate of Hypernatremia Correction and Outcomes in Hospitalized Patients
Date of Completion: March 12, 2025
Disclosure Updated Date: March 12, 2025
