# Supplementary material for: Relationship between Rate of Hypernatremia Correction and Outcomes in Hospitalized Patients
Source: Kidney360. 2025 Mar 28;6(8):1305–16. doi: 10.34067/KID.0000000785 (PMC12407122; doi:10.34067/KID.0000000785)
Supplement: Supplementary file 2 [file kidney360-6-1305-s002.pdf]

Supplemental Material

- **Supplemental Table 1**: **Adjusted SOFA score.** SOFA: Sequential Organ Failure Assessment. MAP: Mean arterial pressure. Patients with non-invasive mechanical ventilation include only those with acute (+/- chronic) respiratory failure or distress.
- **Supplemental Table 2: Relationship between sodium correction rate and disposition outcomes stratified by age.** Slow: ≤0.50 mEq/L/hour; Fast: >0.50 mEq/L/hour. Adjusted odds ratios (aORs) derived from a multinomial logistic regression model adjusted for age, gender, race, Quan-Charlson Comorbidity Index, and adjusted Sequential Organ Failure Assessment (SOFA) score. Propensity scores (PS) included in model as weights. CI: confidence interval.
- **Supplemental Table 3: Relationship between sodium correction rate and disposition outcomes stratified by estimated glomerular filtration rate.** Slow: ≤0.50 mEq/L/hour; Fast: >0.50 mEq/L/hour. Adjusted odds ratios (aORs) derived from a multinomial logistic regression model adjusted for age, gender, race, Quan-Charlson Comorbidity Index, and adjusted Sequential Organ Failure Assessment (SOFA) score. Propensity scores (PS) included in model as weights. eGFR: estimated glomerular filtration rate. CI: confidence interval.
- **Supplemental Figure 1: Distribution of initial serum sodium concentration in the final cohort.** Sodium concentrations in mEq/L**.**
- **Supplemental Figure 2: Crude probability of discharge to home (a), in-hospital mortality (b), nursing facility (c), and hospice (d) as a function of age stratified by sodium correction rate.** Predicted probabilities derived from a multinomial logistic regression model. Slow: ≤0.50 mEq/L/hour; Fast: >0.50 mEq/L/hour.
- **Supplemental Figure 3: Plots of adjusted odds ratios (aORs) (95% confidence interval) for in-hospital mortality and discharge to hospice or a nursing facility associated with different sodium correction rates stratified by age.** Slow: ≤0.50 mEq/L/hour; Fast: >0.50 mEq/L/hour.

Adjusted odds ratios (aORs) derived from a multinomial logistic regression model adjusted for age, gender, race, Quan-Charlson Comorbidity Index, and adjusted Sequential Organ Failure Assessment (SOFA) score. Model includes propensity scores as weights. <= denotes ≤.

- **Supplemental Figure 4: Crude probability of discharge to home (a), in-hospital mortality (b), nursing facility (c), and hospice (d) as a function of estimated glomerular filtration rate (eGFR) stratified by sodium correction rate.** Predicted probabilities derived from a multinomial logistic regression model. Slow: ≤0.50 mEq/L/hour; Fast: >0.50 mEq/L/hour.
- **Supplemental Figure 5: Plots of adjusted odds ratios (aORs) (95% confidence interval) for in-hospital mortality and discharge to hospice or a nursing facility associated with different sodium correction rates stratified by estimated glomerular filtration rate (eGFR) level.** Slow: ≤0.50 mEq/L/hour; Fast: >0.50 mEq/L/hour Adjusted odds ratios (aORs) derived from a multinomial logistic regression model adjusted for age, gender, race, Quan-Charlson Comorbidity Index, and adjusted Sequential Organ Failure Assessment (SOFA) score. Model includes propensity scores as weights. <= denotes ≤.

Supplemental Table 1. Adjusted SOFA score		
	**Variable**	**Points**
**Respiratory**	All others	0
	Non-invasive mechanical ventilation	2
	Invasive mechanical ventilation for <96 hours	3
	Invasive mechanical ventilation for ≥96 hours	4
**Coagulation:** Platelets x10^3/μL	>150	0
	101-150	1
	51-100	2
	21-50	3
	≤20	4
**Neurologic:** Glasgow Coma Score	15	0
	13-14	1
	10-12	2
	6-9	3
	<6	4
**Liver:** Bilirubin (mg/dL)	<1.2	0
	1.2–1.9	1
	2.0–5.9	2
	6.0–11.9	3
	≥12.0	4
**Cardiovascular:** MAP or Vasopressor Administration	MAP ≥70 mmHg without use of vasopressor agents	0
	MAP <70 mmHg without use of vasopressor agents	1
	Any use of dopamine or dobutamine	2
	Any use of norepinephrine	3
	Use of norepinephrine and vasopressin	4
**Renal:** Creatinine (mg/dL)	<1.2	0
	1.2–1.9	1
	2.0–3.4	2
	3.5–4.9	3
	≥5.0	4

SOFA: Sequential Organ Failure Assessment. MAP: Mean arterial pressure. Patients with non-invasive mechanical ventilation include only those with acute (+/- chronic) respiratory failure or distress.

**Supplemental Table 2. Relationship between sodium correction rate and disposition outcomes stratified by age**				
**Age Group**	**Correction Rate**	**In-Hospital Mortality (n = 4,285)**	**Discharge to Hospice (n = 1,258)**	**Discharge to Nursing Facility (n = 15,256)**
		**PS Weighted**	**PS Weighted**	**PS Weighted**
		aOR (95% CI)	aOR (95% CI)	aOR (95% CI)
**18-45**	**Slow**	0.58 (0.45, 0.74)	1.35 (0.54, 3.34)	1.99 (1.52, 2.61)
	**Fast**	Reference	Reference	Reference
**46-65**	**Slow**	0.81 (0.66, 0.98)	3.61 (1.73, 7.53)	6.36 (5.04, 8.01)
	**Fast**	1.32 (1.09, 1.58)	3.11 (1.48, 6.53)	3.76 (2.97, 4.76)
**66-75**	**Slow**	1.67 (1.36, 2.04)	12.70 (6.19, 26.06)	16.77 (13.29, 21.17)
	**Fast**	2.87 (2.38, 3.46)	4.79 (2.26, 10.15)	10.40 (8.22, 13.16)
**76-89**	**Slow**	3.50 (2.92, 4.20)	29.50 (14.60, 59.59)	37.67 (29.98, 47.34)
	**Fast**	5.31 (4.46, 6.33)	18.69 (9.22, 37.88)	24.78 (19.72, 31.15)
**90+**	**Slow**	7.13 (5.70, 8.92)	67.57 (32.89, 138.83)	68.51 (53.31, 88.06)
	**Fast**	11.10 (9.03, 13.64)	50.47 (24.56, 103.74)	40.38 (31.47, 51.82)

Slow: ≤0.50 mEq/L/hour; Fast: >0.50 mEq/L/hour. Adjusted odds ratios (aORs) derived from a multinomial logistic regression model adjusted for age, gender, race, Quan-Charlson Comorbidity Index, and adjusted Sequential Organ Failure Assessment (SOFA) score. Propensity scores (PS) included in model as weights. CI: confidence interval.

**Supplemental Table 3. Relationship between sodium correction rate and disposition outcomes stratified by estimated glomerular filtration rate**				
**eGFR**	**Correction Rate**	**In-Hospital Mortality (n = 4,285)**	**Discharge to Hospice (n = 1,258)**	**Discharge to Nursing Facility (n = 15,256)**
		**PS Weighted**	**PS Weighted**	**PS Weighted**
		aOR (95% CI)	aOR (95% CI)	aOR (95% CI)
**0-14**	**Slow**	0.43 (0.35, 0.54)	1.65 (1.08, 2.52)	1.24 (1.05, 1.47)
	**Fast**	0.71 (0.58, 0.86)	1.35 (0.89, 2.06)	0.69 (0.58, 0.82)
**15-29**	**Slow**	0.83 (0.70, 1.00)	2.16 (1.49, 3.14)	1.88 (1.64, 2.15)
	**Fast**	1.59 (1.35, 1.89)	2.29 (1.59, 3.32)	1.29 (1.12, 1.49)
**30-59**	**Slow**	0.86 (0.74, 1.00)	1.68 (1.19, 2.38)	1.63 (1.47, 1.82)
	**Fast**	1.60 (1.39, 1.85)	0.93 (0.64, 1.35)	1.05 (0.94, 1.17)
**60-89**	**Slow**	0.94 (0.81, 1.11)	2.01 (1.42, 2.84)	1.47 (1.32, 1.64)
	**Fast**	1.28 (1.11, 1.49)	1.03 (0.71, 1.48)	0.92 (0.82, 1.02)
**90+**	**Slow**	0.77 (0.65, 0.92)	2.76 (1.90, 4.00)	1.56 (1.39, 1.75)
	**Fast**	Reference	Reference	Reference

Slow: ≤0.50 mEq/L/hour; Fast: >0.50 mEq/L/hour. Adjusted odds ratios (aORs) derived from a multinomial logistic regression model adjusted for age, gender, race, Quan-Charlson Comorbidity Index, and adjusted Sequential Organ Failure Assessment (SOFA) score. Propensity scores (PS) included in model as weights. eGFR: estimated glomerular filtration rate. CI: confidence interval.

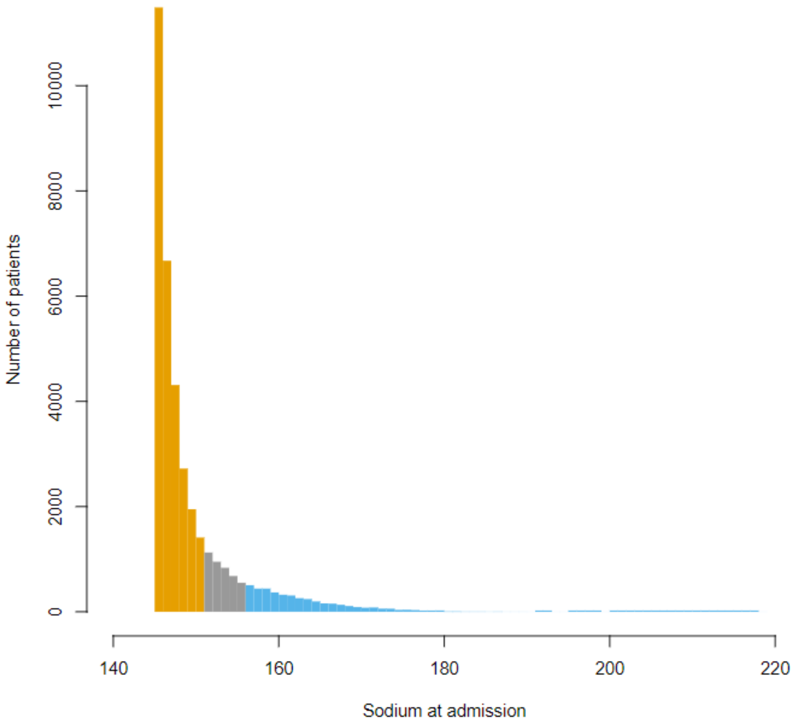

Supplemental Figure 1: Distribution of initial serum sodium concentrations in the final cohort. Sodium concentrations in mEq/L.

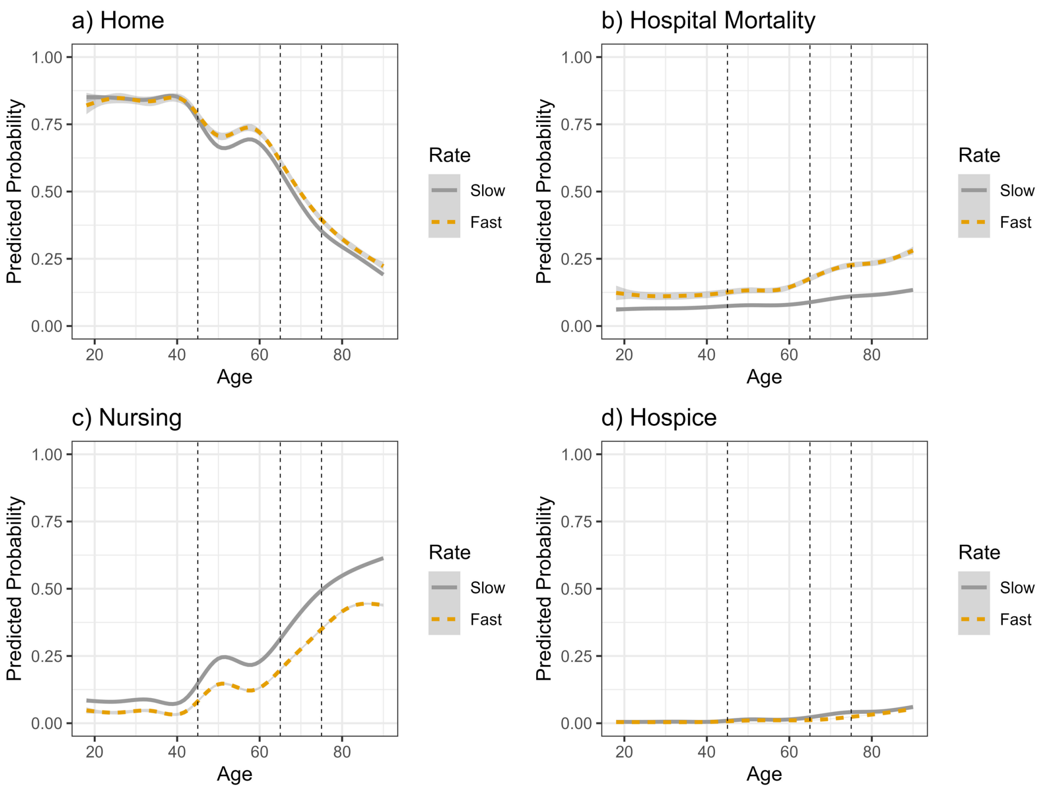

Supplemental Figure 2. Crude probability of discharge to home (a), in-hospital mortality (b), nursing facility (c), and hospice (d) as a function of age stratified by sodium correction rate. Predicted probabilities derived from a multinomial logistic regression model. Slow: ≤0.50 mEq/L/hour; Fast: >0.50 mEq/L/hour.

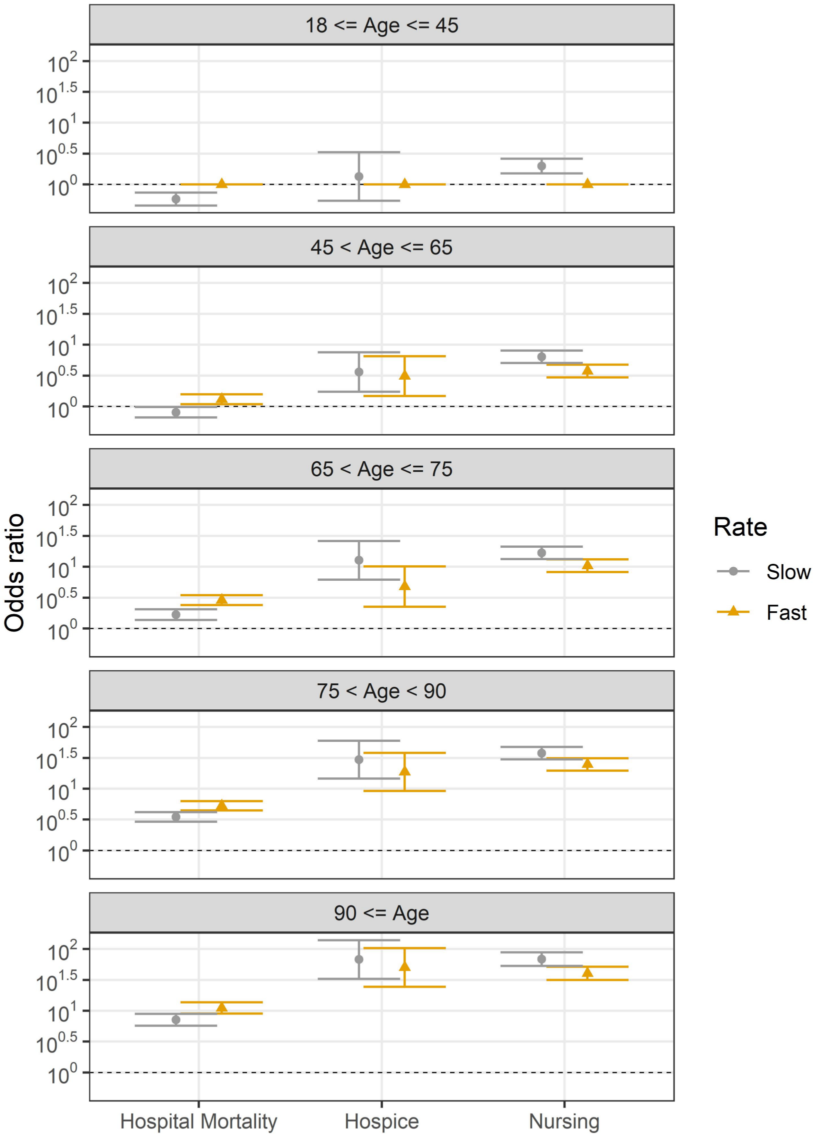

Supplemental Figure 3. Plots of adjusted odds ratios (aORs) (95% confidence interval) for in-hospital mortality and discharge to hospice or a nursing facility associated with different sodium correction rates stratified by age. Slow: ≤0.50 mEq/L/hour; Fast: >0.50 mEq/L/hour. Adjusted odds ratios (aORs) derived from a multinomial logistic regression model adjusted for age, gender, race, Quan-Charlson Comorbidity Index, and adjusted Sequential Organ Failure Assessment (SOFA) score. Model includes propensity scores as weights. <= denotes ≤.

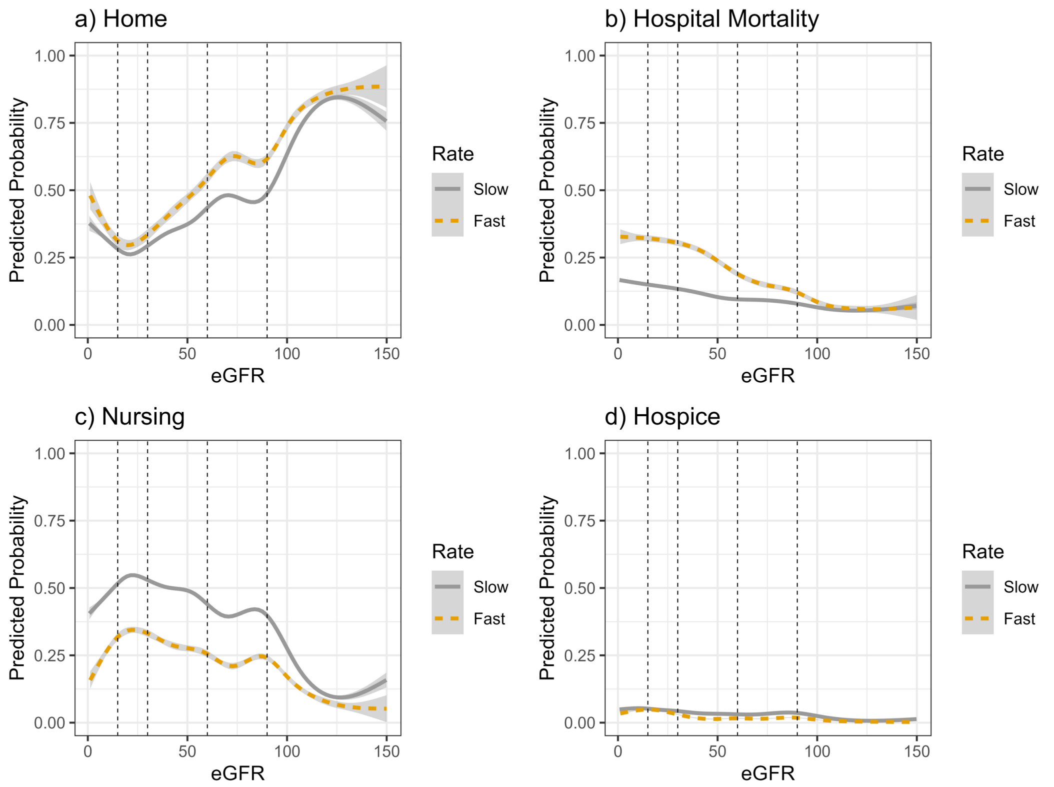

Supplemental Figure 4. Crude probability of discharge to home (a), in-hospital mortality (b), nursing facility (c), and hospice (d) as a function of estimated glomerular filtration rate (eGFR) stratified by sodium correction rate. Predicted probabilities derived from a multinomial logistic regression model. Slow: ≤0.50 mEq/L/hour; Fast: >0.50 mEq/L/hour.

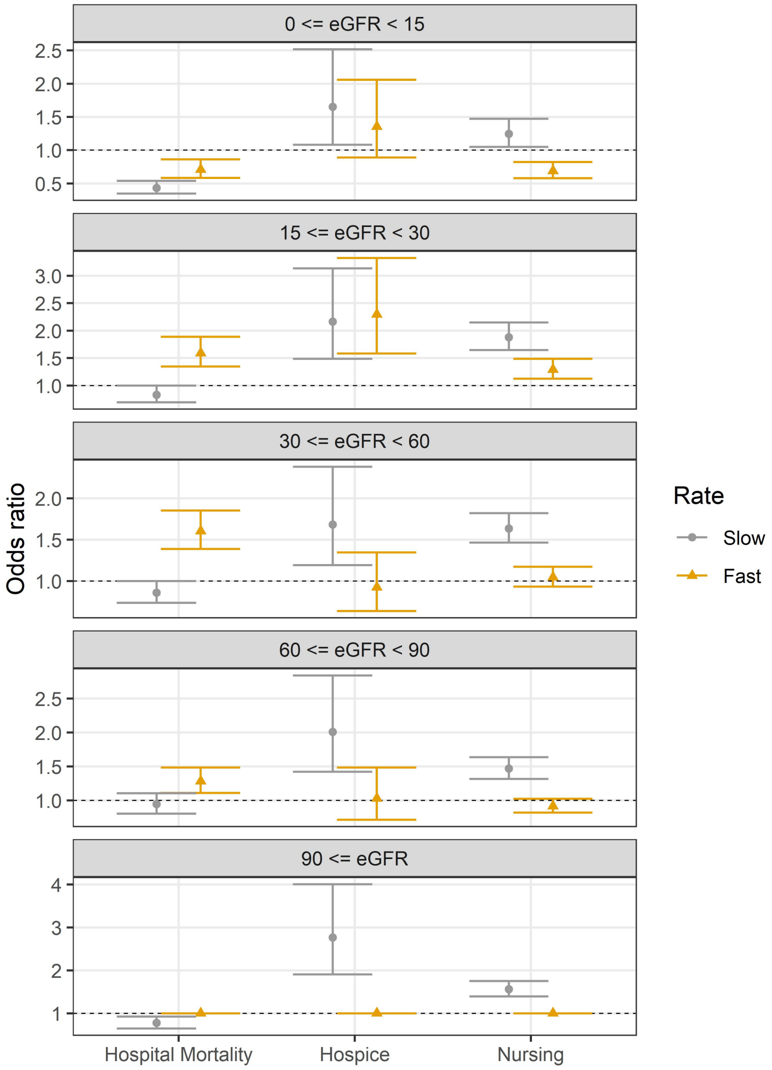

Supplemental Figure 5. Plots of adjusted odds ratios (aORs) (95% confidence interval) for in-hospital mortality and discharge to hospice or a nursing facility associated with different sodium correction rates stratified by estimated glomerular filtration rate (eGFR) level. Slow: ≤0.50 mEq/L/hour; Fast: >0.50 mEq/L/hour Adjusted odds ratios (aOR) derived from a multinomial logistic regression model adjusted for age, gender, race, Quan-Charlson Comorbidity Index, and adjusted Sequential Organ Failure Assessment (SOFA) score. Model includes propensity scores as weights. <= denotes ≤.
